# Population Structure and Oxacillin Resistance of *Staphylococcus aureus* from Pigs and Pork Meat in South-West of Poland

**DOI:** 10.1155/2015/141475

**Published:** 2015-05-03

**Authors:** Paweł Krupa, Jarosław Bystroń, Magdalena Podkowik, Joanna Empel, Aneta Mroczkowska, Jacek Bania

**Affiliations:** ^1^Department of Food Hygiene and Consumer Health Protection, Wrocław University of Environmental and Life Sciences, 50-375 Wroclaw, Poland; ^2^Department of Epidemiology and Clinical Microbiology, National Medicines Institute, 30/34 Chełmska, 00-725 Warsaw, Poland

## Abstract

The genotypes and oxacillin resistance of 420 *S. aureus* isolates from pigs (*n* = 203) and pork (*n* = 217) were analyzed. Among 18 *spa* types detected in *S. aureus* from pig t011, t021, t034, t091, t318, t337, and t1334 were the most frequent. Among 30 *spa* types found in *S. aureus* isolates from pork t084, t091, t499, t4309, t12954, and t13074 were dominant. The animal *S. aureus* isolates were clustered into MLST clonal complexes CC7, CC9, CC15, CC30, and CC398 and meat-derived isolates to CC1, CC7, and CC15. Thirty-six MRSA were isolated exclusively from pigs. All MRSA were classified to *spa* t011 SCC*mec*V. BORSA phenotype was found in 14% *S. aureus* isolates from pigs and 10% isolates from pork meat. *spa* t034 dominated among BORSA from pigs and t091 among meat-derived BORSA. This is the first report on *spa* types and oxacillin resistance of *S. aureus* strains from pigs and pork meat in Poland. Besides *S. aureus* CC9, CC30, and CC398 known to be distributed in pigs, the occurrence of genotype belonging to CC7 in this species has been reported for the first time. To our knowledge it is also the first report concerning CC398 BORSA isolates from pigs and pork meat.

## 1. Introduction

*Staphylococcus aureus* is one of the most serious pathogens of humans and important animal pathogen.* S. aureus* infections can easily turn into life-threatening diseases if they are not antibiotically treated. The ability of this microorganism to survive in the presence of *β*-lactam antibiotics remains the main problem in the therapy [1]. Several phenotypes of resistance to *β*-lactams have been described in* S. aureus* so far. These phenotypes reflect different mechanisms of resistance and include acquisition of *β*-lactamase, modification of normal penicillin-binding proteins (PBPs), and acquisition of genes coding for low-drug-affinity PBPs. Methicillin-resistant* S. aureus* (MRSA), representing the latest of these aforementioned mechanisms, show resistance to both cefoxitin and oxacillin, which is conferred by acquisition of the* mec*A or recently discovered* mec*C gene [2, 3]. Another relatively frequently described phenotype amongst* S. aureus* strains is borderline oxacillin-resistant* S. aureus* (BORSA). These strains are cefoxitin susceptible and do not carry the* mec*A or* mec*C genes but are characterized by oxacillin resistance with MIC between 1 and 8 *μ*g/mL [2, 3]. Hyperproduction of *β*-lactamase has been proposed to explain BORSA phenotype [4–6].

Increasing number of community-acquired MRSA (CA-MRSA) infections led to investigation of new sources of their origin. Current knowledge on population structure of cefoxitin-susceptible* S. aureus* from animals is still scarce. Some* S. aureus* genotypes are thought to be predominantly associated with particular animal species. The others can be isolated from both human and animals [7, 8]. Most of available data concern prevalence of animal MRSA [9–12]. Evidence for multiple, independent acquisition of the methicillin resistance determinant of methicillin-susceptible* S. aureus* (MSSA) strains, associated with animal breeding, enforces the research on revealing the structure of this population. Some of* S. aureus *lineages observed in animals are thought to arise from relatively recent transmission from humans. They may represent important reservoir of strains affecting global health systems. Research on genetic diversity of animal MSSA may allow identification of new clones potentially contributing to emergence of community-acquired staphylococcal infections [7].

Until recently MRSA were rarely isolated from livestock animals. However, in recent years, livestock-associated MRSA have been repeatedly isolated from pigs initially in Netherlands [13] and later in various countries in Europe [10], Canada [14], and USA [11]. Lineage ST398 was found to predominate among MRSA in pigs in Europe. It was observed that MRSA CC398 can be readily transferred from animal to animal and animal to human. Studies in humans showed that rapid transmission of MRSA CC398 is possible even after short-term occupational contact with colonized pigs [15].

*spa* genotyping scheme, first introduced by Harmsen et al. [16], enables interlaboratory comparison of genotypic data. Its association to the results of MLST typing allows for reliable genotypic characterization of* S. aureus* populations [17].

The aim of this work was to determine the population structure of* S. aureus* isolates derived from pig carriage and porcine meat, based on* spa* typing, with emphasis on detection of oxacillin-resistant isolates, that is, BORSA and MRSA.

## 2. Materials and Methods

### 2.1. Isolation and Identification of* S. aureus*

One thousand and seventy-four nasal swabs were taken between 2011 and 2012 from pigs in two slaughterhouses (S1 and S2) possessing their own meat processing plants located in south-west of Poland. Eight hundred and four nasal swabs were taken from the slaughterhouse S1 and 270 from the slaughterhouse S2. Average slaughter capacity was 200 pigs per day in both plants. The swabs were taken (ca. one hundred swabs per sampling) four times from slaughterhouse S1 and two times from S2 in 2011, as well as four times from S1 and once from S2 in 2012. Animals at each sampling session originated from different farms (11 objects in total). Samples were collected from the nasal cavity by introducing a cotton swab for approximately 10 cm into the nares. The swabs were taken after electric stunning, before steaming of the pigs. Slaughtered animals originated from local breeding farms.

Additionally, 396 and 140 samples of pork meat from S1 and S2 company shops were examined, respectively. Meat samples (ca. 20) were purchased from the company shops everyday successively during maximum of 4 days following the slaughter.

One-gram food samples and whole nasal cotton swabs were cultured in a final volume of 10 mL of Giolitti-Cantoni enrichment broth and subcultured on Baird-Parker agar. The isolates were identified as* S. aureus *based on their ability to coagulate rabbit plasma and clumping factor production. All isolates were screened by PCR using* S. aureus*-specific primers for* nuc *gene, encoding thermonuclease [18]. Reference* S. aureus* strain ATCC 29213 served as a control. One* S. aureus* isolate per sample/swab was taken for further characterization.

### 2.2. Preparation of Bacterial DNA

Two millilitres of bacterial cell suspension from an overnight culture grown in brain-heart infusion (BHI) broth was centrifuged for 5 min at 12,000 ×g and suspended in 100 *μ*L of 100 mM Tris-HCl buffer, pH 7.4, containing 10 *μ*g of lysostaphin (A&A Biotechnology, Gdańsk, Poland). After 30-minute incubation at 37°C, 10 *μ*L of 10% SDS was added and the sample was incubated for another 30 min at 37°C. Two hundred *μ*L of 5 M guanidine hydrochloride was added and the sample was mixed by vortexing and incubated at room temperature for 10 min. The DNA was extracted by phenol and chloroform, precipitated with ethanol, and dissolved in water.

### 2.3. Detection of* mec*A and Determination of SCC*mec* Cassette Type

All* S. aureus* isolates were tested for the presence of* mec*A gene using the primers described by Milheiriço et al. [19]. Each PCR contained* mec*A-positive (*S. aureus* ATCC 43300) and -negative (*S. aureus* ATCC 29213) strains as controls. SCC*mec* cassette type was determined according to Milheiriço et al. [19]. The PCR products were electrophoretically resolved in 1.5% agarose containing 0.5 *μ*g/mL ethidium bromide and photographed with the GelDocXR System (Bio-Rad, Hercules, CA).

### 2.4. Antibiotic Resistance and Oxacillin MIC Determination in* mec*A-Positive* S. aureus*

Susceptibility of* mec*A-positive* S. aureus* isolates to penicillin G (10 units/disc), cefoxitin (30 *μ*g/disc), tetracycline (30 *μ*g/disc), clindamycin (2 *μ*g/disc), gentamicin (10 *μ*g/disc), erythromycin (15 *μ*g/disc), ciprofloxacin (5 *μ*g/disc), norfloxacin (10 *μ*g/disc), and vancomycin (30 *μ*g/disc) (all substances from Oxoid Ltd., UK) was tested by the disk-diffusion method and interpreted according to CLSI document M100-S22 [20]. The MIC for oxacillin was determined with the *E*-test and interpreted according to the manufacturer's instructions (bio-Mérieux, Inc.). Reference* S. aureus* strains ATCC 25923, ATCC 43300, and ATCC 29213 served as controls.

### 2.5. Detection of Borderline Oxacillin-Resistant* S. aureus* (BORSA)

All* mec*A-negative* S. aureus* isolates were plated on oxacillin resistance screening agar (ORSA, Oxoid) plates containing 2, 3, and 4 *μ*g/mL oxacillin, respectively. The results were recorded after 24- and 48-hour incubation at 35°C and interpreted according to the manufacturer's instructions. For isolates able to grow in medium containing 4 *μ*g/mL oxacillin MIC for oxacillin was determined using *E*-test (bio-Mérieux, Inc.). Reference MRSA (ATCC 43300) and MSSA (ATCC 29213) strains served as controls. All phenotypically oxacillin-resistant isolates were analyzed for susceptibility to amoxicillin with clavulanic acid (20/10 *μ*g/disc) by the disk-diffusion method and interpreted according to CLSI document M100-S22 [20]. Reference* E. coli* ATCC 35218 strain served as control. All BORSA isolates were screened for* blaZ* gene according to Rizzotti et al. [21] and for hyperproduction of beta-lactamase using Cefinase test (bio-Mérieux, Inc.).

### 2.6. Detection of* mec*C

All* mec*A-negative* S. aureus* isolates able to grow on ORSA plates containing 2 *μ*g/mL oxacillin were tested for* mec*C gene using the primers described by Cuny et al. [22]. DNA from* mec*C-positive* S. aureus *strain 1140/12, from the National Medicines Institute, Warsaw, Poland, served as a control.

### 2.7. Determination of* spa* Type and ST

The* spa* types of all* S. aureus* isolates were determined according to Harmsen et al. [16]. The nucleotide sequencing of the repeat-containing region of the* spa* gene was performed from both DNA strands of the PCR product by Genomed (Warsaw, Poland), using BigDye Terminator Ready Reaction Cycle Sequencing kit. The analysis of repeats and the assignment of* spa* types were performed with the resources of the Ridom SpaServer (http://spa.ridom.de). Grouping of* spa* types was done using BURP, Ridom Staphtype Software.* Spa* types were clustered if cost between members of the group was less than or equal to 4.* Spa* types shorter than 5 repeats were excluded from analysis [23].

Sequence types (STs) of selected* S. aureus* isolates (one t091 isolate from pig and 9 isolates from meat, i.e., t091, t118, t289, t519, t3358, t9031, t12953, t12954, and t12955) were determined according to Enright et al. [24]. The sequences obtained from both strands of the PCR product were analyzed using BioEdit software (http://www.mbio.ncsu.edu/bioedit/bioedit.html) and further assignment of the sequence type (ST) was performed using the http://www.mlst.net/ platform.

## 3. Results

### 3.1. Frequency of* S. aureus* Isolates

In total, 420* S. aureus* isolates were obtained, including 203 isolates from 1074 nasal swabs and 217 isolates from 536 meat samples. The prevalence of* S. aureus* was different in the two slaughterhouses. The bacterium was found in 197 (25%) from a total of 804 nasal swabs in S1, but only in 6 (2%) from 270 swabs in S2. Screening of meat from S1 and S2 company shops revealed comparable prevalence of samples contaminated with* S. aureus*. The pathogen was isolated from 157 (40%) of a total of 396 meat samples derived from S1 and 60 (43%) from 140 samples originating from S2.

### 3.2. Genotypes of * S. aureus* Isolates

Forty-three* spa* types were determined in studied* S. aureus* population.* S. aureus* isolates obtained from pig nasal swabs were classified into 18* spa* types. Among them t318 (28.7%), t011 (18.3%), t034 (13.4%), t337 (11.9%), t021 (8.9%), t091 (8.4%), and t1334 (3.0%) were most frequent (Table 3), with genotypes t318, t034, and t091 isolated during 5 out of a total of 11 sampling sessions (Tables 1 and 2).

*S. aureus* isolates from pork meat were assigned to 30* spa* types. Among them isolates belonging to t091 (41.7%), t4309 (14.2%), t084 (11.5%), t499 (5.5%), t12954 (5.5%), and t13074 (3.7%) were dominating (Table 3).* S. aureus* genotype t091 was isolated during 9 out of 11 sampling sessions, while t4309 was found in samples from 5 sessions (Tables 1 and 2).

The most abundant genotype amongst isolates from animals, t318 and t011, were not found in food (Tables 1 and 2).* spa* genotypes t034 and t337 were frequently isolated from animals (13.3% and 11.8%, resp.), but sporadically from meat (1.8% and 0.9%, resp.). In contrast, genotypes t4309 and t084 occurred sporadically in pigs (0.5% each) but were frequent in pork meat (14.3% and 11.5%, resp.) (Tables 1 and 2). Thirteen out of 18* S. aureus* genotypes identified in animals were not detected in meat, whereas among 30* spa* types found in meat 25 were not detected in animals. Only 5* S. aureus* genotypes, that is, t034, t084, t091, t337, and t4309, were identified in both nasal swabs and meat (Table 3).

**Figure 1:**
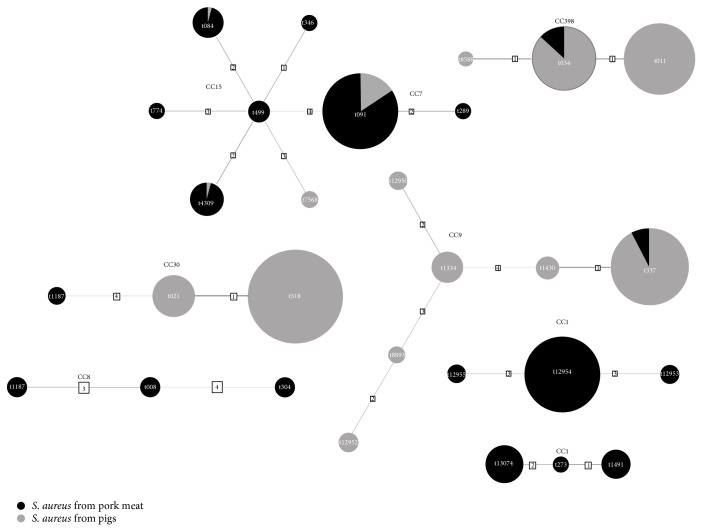
Cluster analysis of* spa* types of* S. aureus* isolates from pig nasal swabs and pork meat.

All genotypes identified in the studied* S. aureus* isolates were clustered into 7* spa* complexes (Figure 1). The animal isolates were clustered into four complexes, namely,* spa*-CC034 (t011, t034, and t8588) belonging to ST-CC398,* spa*-CC1334 (t337, t1334, t1430, t8893, t12950, and t12952) within ST-CC9,* spa*-CC021 (t021, t318) within ST-CC30, and* spa*-CC499 (t084, t091, t4309, and t7568) within ST-CC7 and ST-CC15 (Figure 1). The* spa* genotypes of meat-derived* S. aureus* isolates were clustered into 7 complexes. Most isolates (73.4%) were grouped into* spa*-CC499 belonging to ST-CC15 and ST-CC7. Other numerous complexes included* spa*-CC273 (7.8%) and newly described* spa*-CC12954 (6.4%) both belonging to ST-CC1 (Figure 1, Table 3).

### 3.3. Characterization of MRSA Isolates

Thirty-six (17.8%)* S. aureus* isolates from pigs were classified as MRSA. All these isolates were* mec*A-positive and resistant to cefoxitin in the disc-diffusion method. None of them possessed* mec*C gene. All of the isolates were resistant to oxacillin with MIC ranged from 32 to 48 *μ*g/mL. Additionally all of the MRSA isolates were resistant to penicillin and tetracycline and susceptible to gentamicin, erythromycin, ciprofloxacin, norfloxacin, and vancomycin. MRSA were isolated exclusively from pigs in slaughterhouse S1, originating from a single farm. All of them were classified to* spa* type t011 SCC*mec*V.

### 3.4. Characterization of BORSA Isolates

Twenty-eight (14%)* S. aureus* isolates from pigs and 21 (10%) from meat exhibited borderline resistance to oxacillin. All of them were* mec*A and* mec*C negative, susceptible to cefoxitin and amoxicillin with clavulanic acid, and able to grow on ORSA plates containing minimum of 2 *μ*g/mL oxacillin. Seventeen (30%) and 3 (5%) of BORSA isolates grew on 3 and 4 *μ*g/mL oxacillin, respectively. MIC for oxacillin was <5 *μ*g/mL in isolates able to grow on 4 *μ*g/mL oxacillin. All BORSA isolates were positive in Cefinase test and harboured* blaZ* gene.* spa* t034 was found to dominate among BORSA from pigs (64%) and t091 among meat-derived BORSA (38%) (Table 4).

## 4. Discussion

Animal production models, that is, concentration of production in limited number of big holdings or in numerous small farms, are thought to influence the structure of* S. aureus* population [7]. Large European screening of pooled dust samples from pig breeding farms which focused on MRSA demonstrated considerable variation in terms of MRSA prevalence and their genotypes among EU countries [10]. Our previous research indicated low incidence of oxacillin-resistant* S. aureus* in food of animal origin in Poland [25]. This encouraged us to investigate the incidence and genotypes of* S. aureus* in pigs and pork meat in south-western Poland.

Our data indicate 19% mean incidence of* S. aureus* in pigs and 40% in pork meat. However, it should be stressed that depending on sampling session it varied from 0% to 48% in animals and from 0 to 44% in meat indicating significant variation in* S. aureus* occurrence among farms. As yet most research on incidence of* S. aureus* in pigs was focussed on MRSA [7]. Studies, like that by Vandendriessche et al. [26], carried out in Belgium, demonstrating MSSA occurrence (27%) and* spa* genotype structure among pigs are still rare.

*S. aureus* genotypes from pigs studied here clustered into four clonal complexes, namely, CC30 (38% of all animal isolates), CC398 (32%), CC9 (19%), and CC7 (8%). High prevalence of MSSA from genetic lineages CC30 and CC398 has been already reported in pigs in Europe [26, 27]. Animal contamination with ST398 MRSA increased over last years. A number of evidences demonstrate the possibility of their transmission to humans [28]. Data from EFSA report indicate t011 MRSA as most frequent in Europe [10]. Similarly our MRSA isolates of* spa* type t011 constituted most numerous populations within CC398, but it should be emphasized that they were found at one sampling only. According to the only data on CC398 MRSA associated with pig environment in Poland their prevalence was not higher than 2% [10]. Here we demonstrate that although overall prevalence of genotype t011 MRSA in pigs was ca. 3%, their occurrence was likely restricted to single source. In turn, t034 MSSA was the most prevalent genotype (32%) belonging to CC398 in Denmark [27]. Our t034 MSSA isolates, although less numerous (12%), consistently occurred in a series of sessions, what may indicate wide dissemination of this genotype in Poland.

Transmission of CC398 from animals to food was not extensively investigated as yet. CC398 MRSA was already detected in milk and meat [28, 29]. According to some surveys pork meat contamination with CC398 strains was relatively frequent. Results of a Dutch survey report MRSA t011 isolates in 7% of pork meat [28]. Spanish report indicates 3% frequency of ST398 MRSA in raw pork [30]. We could not detect MRSA in pork meat; however our previous surveys on MRSA incidence in food of animal origin support very low frequency of these strains in Poland [25, 31]. Results presented here demonstrated, however, that MSSA and BORSA t034 isolates could be found in pork meat indicating potential of sporadic introduction of animal-associated genotypes into food chain.

Although the incidence of BORSA among human clinical isolates was reported to be about 5%, they have been implicated in community-acquired and hospital infections [32–34]. BORSA have already been detected in food primarily associated with ruminant milk [25, 35]. In turn, cows seem to be the only animal species in which incidence of BORSA was reported [36]. Genotypic structure of human BORSA population is largely unknown, and there are no data on animal BORSA genotypes. In the current study, 28 and 21 BORSA isolates from pigs and pork meat were identified, respectively. As much as 71% of animal BORSA isolates and 10% of meat isolates were assigned to CC398. These results illustrate the possibility of transmission of typical animal-associated, oxacillin-resistant* S. aureus* isolates to food. Remaining numerous BORSA genotypes belong to CC9 in animal isolates, as well as to CC1 and CC7 in meat isolates.

Another major* spa* cluster identified in this study within animal* S. aureus* isolates, including t1334, t337, t1430, t8893, t12950, and 12952, belongs to CC9. LA-MRSA and MSSA of CC9 are frequent in livestock in Asia [12, 37, 38]. Some reports confirm their low-rate occurrence in Europe [26, 27, 39]. As yet the only European animal* spa* types identified within ST9 include t1430, found in poultry in Netherlands [40], t337 isolated from pigs in Denmark and Belgium [26, 27], and t4794 MRSA from Italy [39]. In turn,* spa* types t337 and t899, representing CC9, were the main pig-derived MRSA in Thailand [38] and Hong Kong [12].

Incidence of t091 isolates, representing genotype belonging to ST7 (CC7), which consequently occurred in studied here pigs at several sampling sessions has not been already reported in this species, suggesting the possibility of emergence of new genotype in livestock. t091 CA-MSSA together with t084 and t774 belonging to CC15, also found in studied animals, have been isolated only from human as yet [41] and recently found in broilers [26]. On the other side, isolates of CC7 and CC15 predominated in pork meat investigated here, accounting for 73% of total isolates derived from this product, suggesting additional most likely human source of meat contamination.

Isolates belonging to CC1 (almost 14%), including isolates assigned to new* spa*CC 12954 type, and CC8 (1.3%) were detected exclusively in meat. Although sporadic occurrence of these genotypes in livestock was already noted [10, 42, 43] isolation of these clones has been reported mainly from human [41, 44].

Only five genotypes, that is, t091, t034, t337, t084, and t4309, were isolated from both animals and pork meat. From these only t091 was identified with high frequency in both populations, while other genotypes consistently predominated in pigs (t034, t337) or meat (t084, t4309). Taking into account a significant diversity of bacterial genotypes revealed in consecutive samplings we cannot draw definite conclusions on the adaptation of studied genotypes to animal or food milieu. However, results of this survey generally demonstrating a weak genotypic relatedness of* S. aureus* from pigs and isolates from pork meat may indicate that certain genotypes can be introduced more readily than others into food chain. It seems that t091 genotype isolates can be better fitted to colonize both pigs and porcine food products. Although our data showed additional nonanimal source of meat contamination, as discussed above for CC1, CC7, CC8, and CC15 isolates, meat contamination may also be associated with* S. aureus* derived from niches other than pig nares, like skin or intestinal tract. Additional research should be conducted to shed light on structure of* S. aureus* carriage in animals.

## 5. Conclusions

Taking together, we first report on* spa* types and the oxacillin resistance of* S. aureus* isolates from pigs and pork meat in Poland. Besides* S. aureus* CC30, CC398, and CC9 already known to be widely distributed in pigs, genotype t091 belonging to CC7 was first reported to occur in this species. This is also the first report on the occurrence of CC398 BORSA isolates in pigs and MSSA and BORSA CC398 isolates in pork meat. CC7 isolates, including BORSA phenotype, together with isolates assigned to CC15 were shown to dominate in pork meat.

## Figures and Tables

**Table 1:** *spa* types of *S. aureus* isolates from pig nasal swabs and pork meat derived from slaughterhouses S1 and S2 in 2011.

Sampling	Nasal swabs			Pork meat		
	Number of samples	Number of isolates	*spa *types (*n*)	Number of samples	Number of isolates	*spa *types (*n*)
*S. aureus* isolates from S1						
Sampling I	101	44	t318 (29), t034 (7), t1430 (3), t091 (2), t7568 (1), t4309 (1), t8588 (1)	95	32	t091 (12), t084 (12), t4309 (3), t273 (1), t499 (2), t015 (1), t118 (1)
Sampling II	101	19	t021 (15), t318 (3), t034 (1)	65	11	t091 (7), t084 (2), t273 (2)
Sampling III	100	0	—	Not tested	0	—
Sampling IV	100	2	t1334 (2)	36	0	—
Total	**402**	**65**		**196**	**43**	

*S. aureus* isolates from S2						
Sampling I	100	1	t252 (1)	50	21	t091 (6), t084 (4), t034 (2), t499 (2), t156 (1), t289 (1), t304 (1), t346 (1), t519 (1), t1491 (1), t3358 (1)
Sampling II	100	3	t12950 (2), t1430 (1)	50	22	t084 (6), t1491 (5), t091 (4), t3478 (2), t034 (1), t127 (1), t267 (1), t337 (1), t499 (1)
Total	**200**	**4**		**100**	**43**	

**Table 2:** *spa* types of *S. aureus* isolates from pig nasal swabs and pork meat derived from slaughterhouses S1 and S2 in 2012.

Sampling	Nasal swabs			Pork meat		
	Number of samples	Number of isolates	*spa* types (*n*)	Number of samples	Number of isolates	*spa* types (*n*)
*S. aureus* isolates from S1						
Sampling I	101	39	t318 (24), t034 (6), t091 (4), t1334 (4), t1939 (1)	50	26	t4309 (11), t091 (10), t13074 (2), t034 (1), t1187 (1), t9031 (1)
Sampling II	101	49	t011 (37), t091 (4), t021 (3), t034 (2), t026 (1), t084 (1), t1334 (1)	50	31	t091 (9), t4309 (7), t12954 (7), t499 (3), t084 (1), t337 (1), t12955 (1), t13074 (1)
Sampling III	100	32	t337 (21), t034 (9), t091 (1), t318 (1)	50	27	t091 (15), t4309 (7), t13074 (2), t078 (1), t499 (1), t12954 (1)
Sampling IV	100	12	t091 (6), t337 (3), t034 (2), t8893 (1)	50	30	t091 (19), t12954 (4), t4309 (3), t13074 (2), t499 (1), t774 (1)
Total	**402**	**132**		**200**	**114**	

*S. aureus* isolates from S2						
Sampling I	70	2	t318 (1), t12952 (1)	40	17	t091 (9), t499 (2), t3380 (2), t008 (1), t1333 (1), t3358 (1), t12953 (1)
Total	**70**	**2**		**40**	**17**	

**Table 3:** Clonal *spa* complexes of *S. aureus* isolates from pig nasal swabs and pork meat.

Cluster	*spa *	*spa*-CC	MLST-CC	*n*	Origin
1	t091	*spa*-CC 499	CC7^*^	108	Nasal swab (*n* = 17) and pork meat (*n* = 91)
1	t4309	*spa*-CC 499	CC15	32	Nasal swab (*n* = 1) and pork meat (*n* = 31)
1	t084	*spa*-CC 499	CC15	26	Nasal swab (*n* = 1) and pork meat (*n* = 25)
1	t499	*spa*-CC 499	CC15	12	Pork meat
1	t346	*spa*-CC 499	CC15	1	Pork meat
1	t289	*spa*-CC 499	CC7^*^	1	Pork meat
1	t774	*spa*-CC 499	CC15	1	Pork meat
1	t7568	*spa*-CC 499	CC15	1	Nasal swab
2	t337	*spa*-CC 1334	CC9	26	Nasal swab (*n* = 24) and pork meat (*n* = 2)
2	t1334	*spa*-CC 1334	CC9	7	Nasal swab
2	t1430	*spa*-CC 1334	CC9	4	Nasal swab
2	t12950	*spa*-CC 1334	CC9	2	Nasal swab
2	t8893	*spa*-CC 1334	CC9	1	Nasal swab
2	t12952	*spa*-CC 1334	CC9	1	Nasal swab
3	t13074	*spa*-CC 273	CC1	8	Pork meat
3	t1491	*spa*-CC 273	CC1	6	Pork meat
3	t273	*spa*-CC 273	CC1	3	Pork meat
4	t034	*spa*-CC 034	CC398	31	Nasal swab (*n* = 27) and pork meat (*n* = 4)
4	t011	*spa*-CC 034	CC398	37	Nasal swab
4	t8588	*spa*-CC 034	CC398	1	Nasal swab
5	t12954	*spa*-CC 12954	CC1^*^	12	Pork meat
5	t12955	*spa*-CC 12954	CC1^*^	1	Pork meat
5	t12953	*spa*-CC 12954	CC1^*^	1	Pork meat
6	t1333	*spa*-CC 021	CC30	1	Pork meat
6	t318	*spa*-CC 021	CC30	58	Nasal swab
6	t021	*spa*-CC 021	CC30	18	Nasal swab
7	t304	*spa*-CC 008	CC8	1	Pork meat
7	t1187	*spa*-CC 008	CC8	1	Pork meat
7	t008	*spa*-CC 008	CC8	1	Pork meat
Singleton	t3478		CC5	2	Pork meat
Singleton	t3380		CC1	2	Pork meat
Singleton	t3358		CC101^*^	2	Pork meat
Singleton	t015		CC45	1	Pork meat
Singleton	t078		CC25	1	Pork meat
Singleton	t127		CC1	1	Pork meat
Singleton	t156		CC12	1	Pork meat
Singleton	t267		CC97	1	Pork meat
Singleton	t9031		ST1027^*^	1	Pork meat
Singleton	t252		CC15	1	Nasal swab
Excluded	t118		ST2811^*^	1	Pork meat
Excluded	t519		CC7^*^	1	Pork meat
Excluded	t1939		CC398	1	Nasal swab
Excluded	t026		CC45	1	Nasal swab

Total *spa *	43		Total isolates	420	

MLST-CCs marked with asterisk (∗) were determined in this study as described by Enright et al. (2000) [24]. For the remaining MLST-CCs, associations with the particular *spa* types were assessed through the Ridom SpaServer (http://spa.ridom.de) or from the relevant literature.

**Table 4:** Characteristics of BORSA isolates from pig nasal swabs and pork meat.

Oxacillin concentration permitting bacterial growth	Nasal swabs		Pork meat	
	*spa *type	Number of isolates	*spa *type	Number of isolates
4 *μ*g/mL	t034	1	t091	2

3 *μ*g/mL	t034	6	t091	6
	t011	1	t034	1
	t8588	1	t13074	1
	t337	1		

2 *μ*g/mL	t034	11	t12954	4
	t318	1	t273	2
	t337	4	t1491	1
	t026	1	t034	1
	t1334	1	t127	1
			t1187	1
			t13074	1

		Total 28		Total 21
